# Advances in augmenting infiltration of active natural killer cells into pediatric and adult solid tumors

**DOI:** 10.1016/j.omton.2025.201061

**Published:** 2025-09-19

**Authors:** Shiori Eguchi, Wen Luo, Hongwen Zhu, Jeremy M. Rosenblum, Anna R. Cooper, Mitchell S. Cairo

**Affiliations:** 1 Department of Pediatrics, New York Medical College, Valhalla, NY 10595, USA; 2 Department of Pediatrics and Developmental Biology, Institute of Science Tokyo, Tokyo 113-8519, Japan; 3 Department of Pathology, Microbiology and Immunology, New York Medical College, Valhalla, NY 10595, USA; 4 Department of Orthopedic Surgery, New York Medical College, Valhalla, NY 10595, USA; 5 Department of Medicine, New York Medical College, Valhalla, NY 10595, USA; 6 Department of Cell Biology and Anatomy, New York Medical College, Valhalla, NY 10595, USA

**Keywords:** MT: Special Issue - Advancements in pediatric cancer therapy, solid tumors, tumor microenvironment, natural killer cells, chimeric antigen receptor, chemokine receptor, chemokine, anti-TGF-β therapy, immune checkpoint inhibitors, cytokine stimulation, natural killer cell engagers

## Abstract

Although adoptive natural killer cell therapies have been safe and somewhat effective in hematological malignancies, their performance in solid tumors is hindered by the solid tumor microenvironment, which impairs natural killer cell trafficking and function. Strategies that boost natural killer cell infiltration and maintain their activity in the tumor microenvironment have demonstrated enhanced therapeutic benefits. Multiple strategies have been explored to enhance natural killer cell infiltration and sustain their activity within the tumor microenvironment. These encompass tactics like equipping natural killer cells with chemokine receptors or inducing tumor cells to secrete chemokines to enhance homing, arming natural killer cells with chimeric antigen receptors or concomitant use of natural killer cell engagers to direct natural killer cells to tumors, and blocking immunosuppressive factors such as transforming growth factor-β and immune checkpoints or stimulation by cytokines to make the tumor microenvironment more permissive to natural killer cell function, among others. Here we summarize recent advances in the strategies to augment tumor infiltration of active natural killer cells, aiming to improve natural killer cell-based immunotherapies against pediatric and adult solid tumors.

## Graphical abstract

**Figure:**
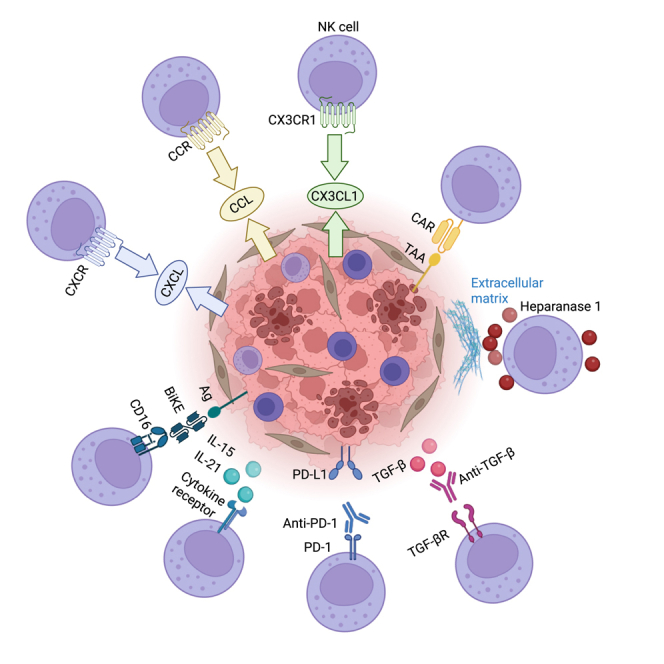

## Teaser

Cairo and colleagues summarized recent advances in strategies to improve infiltration and persistence of active natural killer (NK) cells in the solid tumor microenvironment (TME), including genetic modification of NK cells, manipulation of the TME, cytokine stimulation, and use of NK cell engagers.

## Introduction

Natural killer (NK) cell-based immunotherapies have been demonstrated to be safe and somewhat effective in hematological malignancies. However, these therapies often result in limited *in vivo* expansion or persistence of NK cells, which may contribute to suboptimal long-term efficacy.^1^ In contrast, their success in treating solid tumors has been even more modest. Unlike hematological malignancies, solid tumors exhibit a dense, stroma-rich tumor microenvironment (TME), where interactions between tumor cells and their microenvironment promote tumor progression, invasion, and metastasis.^2^^,^^3^ To elicit effective anti-tumor responses in solid tumors, circulating NK cells must exit the bloodstream, migrate to tumor sites including distant metastatic sites, navigate through the dense extracellular matrix (ECM), and ultimately engage with and respond to tumor cells. Accumulating evidence links higher NK cell presence in the TME with better prognoses across several cancer types, including hepatocellular carcinoma,^4^ melanoma,^5^^,^^6^^,^^7^ breast cancer,^8^ lung cancer,^9^^,^^10^^,^^11^ renal cell carcinoma (RCC),^12^ gastric cancer,^13^ colorectal cancer,^14^ head and neck squamous cell carcinoma (HNSCC),^15^ and neuroblastoma.^16^ However, NK cell infiltration into tumors is limited by insufficient chemotactic signaling and physical barriers within the TME,^17^ and only a few adoptively transferred NK cells successfully reach tumor sites.^18^^,^^19^ Additionally, solid tumor sites are frequently enriched with myeloid-derived suppressor cells, tumor-associated macrophages, and regulatory T cells, which play a crucial role in suppressing NK cell activity. The interaction between tumor cells and stromal cells leads to the release of immunosuppressive molecules such as transforming growth factor (TGF)-β and the increased expression of checkpoint proteins. Together, these factors create a highly immunosuppressive TME that impairs effective NK cell-mediated anti-tumor immune responses.^2^ In this review, we describe strategies developed recently to address these unique challenges in NK cell infiltration associated with the pediatric and adult solid tumor microenvironment.

## Genetic modification of NK cells

### Chemokine receptor-armored NK cells

**Figure 1:**
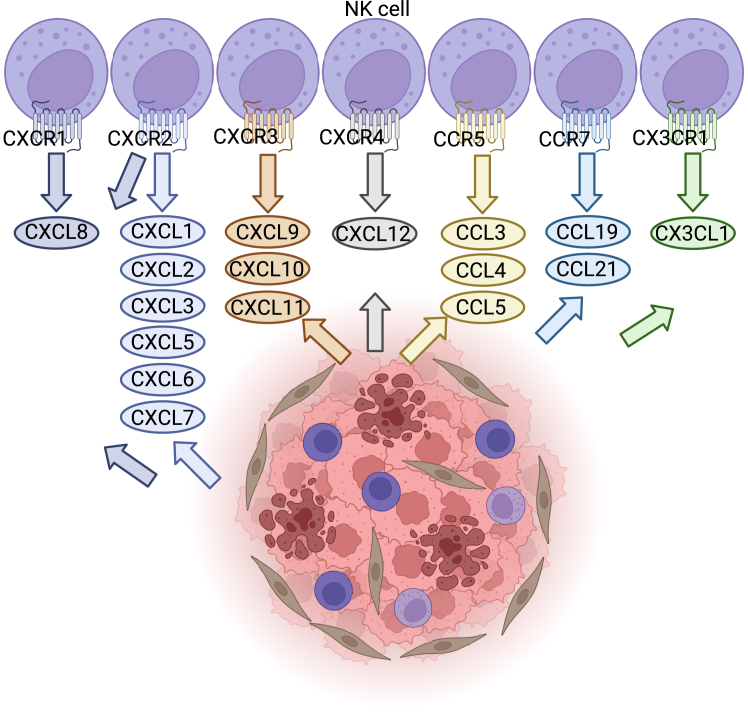
Chemokine control of NK cell migration NK cells are recruited to the TME through chemokine-chemokine receptor signaling pathways. CXCL8 is secreted by some solid tumors and induces chemotaxis through CXCR1 and -2. CXCR2 also induces chemotaxis toward solid tumors secreting CXCL1–3 and CXCL5–7. CXCL12 is secreted by some solid tumors and induces chemotaxis through CXCR4. CCL19 and -21 are secreted by some solid tumors and induce chemotaxis through CCR7. CXCR3 is highly expressed on *ex vivo*-expanded NK cells and induces NK cell migration toward gradients of CXCL9, -10, and -11. CCR5 is expressed at low levels on expanded NK cells and induces NK cell migration toward gradients of CCL3, -4, and -5. CX3CR1 is also expressed on NK cells and induces NK cell migration toward CX3CL1.

Migration of NK cells to tumor tissues occurs when NK-attracting chemokines secreted by tumor cells reach chemokine receptors on NK cells (Figure 1). However, NK cells often do not express receptors recognizing chemokines secreted by tumors, which in part accounts for insufficient NK cell trafficking to tumor sites. Armoring NK cells with a chemokine receptor to chemokines highly secreted by tumors can reverse this chemokine/chemokine receptor mismatch.^20^ Electroporation-induced overexpression of C-X-C motif chemokine receptor (CXCR) 1 markedly improved NK cell infiltration in an HNSCC xenograft model that secretes C-X-C motif chemokine ligand (CXCL) 8. Specifically, by tracking near-infrared fluorescently labeled NK cells, they observed that 72 h after intravenous injection, CXCR1-expressing NK cells accumulated at subcutaneous tumor sites with a signal intensity 10 times higher than that of mock NK cells. In addition, NK cells were electroporated with two mRNA constructs encoding CXCR1 and a chimeric antigen receptor (CAR) targeting NKG2D ligands. The CXCR1-modified NK cells displayed increased migration and infiltration to the tumor, resulting in decreased tumor burden and prolonged survival in mice with ovarian cancer secreting CXCL8.^21^ The modulation of NK cells to express CXCR2 has also demonstrated encouraging results. Retroviral transduction of NK cells to overexpress CXCR2 increased their migration toward RCC cell lines secreting CXCL5 *in vitro*. NK cells expressing CXCR2 exhibited enhanced adhesion capabilities, leading to improved killing of target cells.^22^ Similarly, selection for an enriched CXCR2-positive NK cell population before adoptive transfer demonstrated that CXCR2-positive NK cells have increased infiltration into lung metastases of osteosarcoma.^23^ Alternatively, the CRISPR/Cas9 system was employed to increase the expression of CXCR2 and interleukin (IL)-2 on NK cells, resulting in enhanced migration to tumor sites, greater proliferation, and improved cytotoxicity in a preclinical colon cancer model secreting CXCL1–3 and CXCL5–8.^24^ Combined overexpression of CXCR4 and C-C motif chemokine receptor (CCR) 7 via lentiviral vectors further enhanced NK cell migration in a colon cancer model secreting CXCL12 and C-C motif chemokine ligand (CCL) 21.^25^

Off-target tissue damage is a potential concern in the development of chemokine receptor-armored NK cells. However, although some chemokines (such as CXCL8 and -5) are secreted by inflamed or even healthy tissues,^26^^,^^27^^,^^28^ chemokine receptor (such as CXCR1 and -2)-armored NK cells still require additional activation signals, namely, stress-induced ligands typically found on tumor cells but not on healthy or inflamed tissues, to initiate killing.^29^ Furthermore, NK cells are tightly regulated by inhibitory receptors that help prevent attacks on normal cells.^29^ Therefore, armoring NK cells with chemokine receptors improves NK cell homing to disease sites, while sparing healthy, inflamed areas from unintended NK cell-mediated cytotoxicity. Leveraging this precision, chemokine receptor-armed NK cells can be combined with CARs or NK cell engagers, ensuring NK cells are first brought into the right location and then activated only in the presence of specific tumor antigens.

### CAR NK cells

A CAR is an engineered cell surface receptor that increases the targeting specificity of NK cells and redirects them toward tumor cells expressing the corresponding antigens. A CAR consists of an extracellular domain that recognizes specific antigens and multiple intracellular signaling domains that strongly activate intracellular immune responses upon binding to tumor antigens.^30^ Researchers have developed CAR NK cells directed against multiple solid tumor antigens, including epidermal growth factor receptor (EGFR), human epidermal growth factor receptor 2 (HER2), EGFR variant III (EGFRvIII), GD2, and epithelial cell adhesion molecule (EpCAM), and demonstrated effectiveness in preclinical studies. Schönfeld et al.^31^ engineered NK cells to express a humanized anti-HER2 CAR incorporating CD28 and CD3ζ signaling domains, which efficiently lysed HER2-positive tumor cells *in vitro*. The targeted recognition of tumor cells led to selective accumulation of anti-HER2 CAR NK cells in orthotopic breast cancer xenografts and reduction of lung metastasis in an RCC model. In a separate study,^32^ repeated stereotactic injections of anti-HER2 CAR NK cells prolonged symptom-free survival in mice with glioblastoma xenografts. NK cells were genetically modified to express a second-generation anti-EGFR CAR for targeting breast cancer cells. *In vitro*, these CAR NK cells showed increased cytotoxicity and higher interferon (IFN)-γ production compared with mock NK cells when co-cultured with breast cancer cell lines. In mice with intracranial inoculation of EGFR-expressing breast cancer cells, intratumoral injection of anti-EGFR CAR NK cells significantly reduced tumor growth compared to mock NK cells.^33^ Anti-EGFRvIII CAR NK cells specifically inhibited glioblastoma cell growth by apoptosis in cells expressing EGFRvIII.^34^ Another team^35^ engineered NK cells to stably express an anti-GD2 CAR, enabling these CAR NK cells to effectively recognize and eliminate GD2-expressing neuroblastoma and melanoma cells. Anti-EpCAM CAR NK cells demonstrated strong and selective cytotoxicity against EpCAM-expressing breast cancer cells that were resistant to the natural cytotoxic activity of unmodified NK cells.^36^ We electroporated *ex vivo*-expanded NK cells to express an anti-ROR1 or anti-MCAM CAR and demonstrated the anti-tumor efficacy of these CAR NK cells against neuroblastoma and Ewing sarcoma in *in vitro* and *in vivo* models.^37^^,^^38^^,^^39^^,^^40^ Anti-mesothelin CAR NK cells were derived from induced pluripotent stem cells. These CAR NK cells exhibited strong cytotoxicity against mesothelin-expressing tumors both *in vitro* and *in vivo*, highlighting a promising approach for developing “off-the-shelf” targeted allogeneic cell products for treatment-resistant cancers.^41^

In addition to designing a CAR using the single-chain variable fragment (scFv) of a monoclonal antibody (mAb), a CAR can also be an NK cell-activating receptor-like NKG2D combined with transmembrane and signaling domains. Chang et al.^42^ developed a CAR called NKG2D-DAP10-CD3ζ, which consists of the NK cell-activating receptor NKG2D along with two critical signaling molecules, DAP10 and CD3ζ. NK cells engineered with the NKG2D CAR via retroviral transduction demonstrated significantly increased *in vitro* cytotoxicity against various solid tumor cell lines expressing NKG2D ligands major histocompatibility complex class I chain-related proteins A/B (MICA/B), including the osteosarcoma cell lines U2OS, MG-36, and HOS; the prostate cancer cell lines DU 145 and PC-3; and the rhabdomyosarcoma cell line RH36. Additionally, these CAR NK cells markedly reduced tumor burden in osteosarcoma xenografts in NOD/SCID/gamma^−/−^ (NSG) mice compared with mock NK cells. A similar approach can be used to create other NK cell-activating receptor-based CARs such as an NKp30 CAR to boost NK cell cytotoxicity. The benefit of this CAR approach is that a single CAR can target multiple tumor types that express the corresponding ligands. There are several ongoing or completed clinical trials on CAR NK cells (Table 1).

**Table 1:** Representative clinical trials of CAR NK cells

Therapy	Cancer type	Phase	Status	Trial identifier
Anti-HER2 CAR NK cells	Solid tumors	Phase 1	Completed	NCT04319757
NKG2D CAR NK cells	Solid tumors	Phase 1	Recruiting	NCT05528341
Anti-claudin6/GPC3/mesothelin/AXL CAR NK cells (with IL-7/CCL19 and scFv against PD-1/CTLA-4/Lag-3 secreting vector)	Ovarian, testis, endometrial cancers	Phase 1	Recruiting	NCT05410717
Anti-MICA/B CAR NK cells (with CD38 knockout, high-affinity, non-cleavable CD16R, and IL-15/IL-15Rα fusion protein)	Ovarian, fallopian, primary peritoneal cancers	Phase 1	Recruiting	NCT06342986
Anti-HER2 CAR NK cells + anti-PD-1 mAb	Glioblastoma	Phase 1	Active, not recruiting	NCT03383978
Anti-PD-L1 CAR NK cells	Solid tumors	Phase 1	Active, not recruiting	NCT04050709
Anti-PD-L1 CAR NK cells + N-803 + chemotherapy	Pancreatic cancer	Phase 2	Active, not recruiting	NCT04390399
Anti-PD-L1 CAR NK cells + anti-PD-1 mAb + N-803	Gastric cancer, HNSCC	Phase 2	Active, not recruiting	NCT04847466
Anti-PD-L1 CAR NK cells + anti-PD-1/PD-L1 mAb + N-803	Lung, colorectal, gastric, cervical cancers, melanoma	Phase 2	Recruiting	NCT03228667

CTLA-4, cytotoxic T-lymphocyte associated protein-4; Lag3, lymphocyte-activation gene-3; CD16R, CD16 receptor; IL-15Rα, IL-15 receptor α.

Although CAR NK cell therapies have generally shown lower incidence rates and severity of cytokine release syndrome (CRS) and immune effector cell-associated neurotoxicity syndrome (ICANS) than CAR T cell therapies, emerging reports have identified instances of CRS and ICANS associated with the use of CAR NK cells.^43^^,^^44^ In addition to direct risks to patients and treatment interruption or delay, management of these toxicities often involves immunosuppressive drugs such as corticosteroids, which can dampen NK cell expansion and cytotoxic function, thereby reducing overall anti-tumor efficacy.^45^ Incorporating the inducible caspase 9 (iCasp9) suicide gene into CAR constructs as a “safety switch” has been demonstrated to be efficient in controlling the toxicity of CAR NK cells under unfavorable circumstances.^46^^,^^47^ Mitigation strategies developed for CAR T cell therapies, such as IL-1/IL-6 blockade,^48^ tyrosine kinase inhibitors like dasatinib,^49^ and cytokine modulation strategies including granulocyte-macrophage colony-stimulating factor knockout,^50^ also hold translational promise for CAR NK cell therapies.

## Manipulation of the TME

### Inducing tumor cells to secrete NK-attracting chemokines

Another possible cause of insufficient NK cell infiltration into tumor sites is that tumor cells do not secrete NK-attracting chemokines, while their receptors are readily expressed on NK cells. Manipulation of the TME to secrete chemokine ligands to chemotaxis receptors highly expressed on NK cells can overcome this mismatch.^51^ CXCR3, the receptor for CXCL9, -10, and -11, is present on over 90% of *ex vivo*-expanded NK cells.^52^^,^^53^ IFN-γ induces secretion of CXCR3 ligands in the TME.^54^ Park et al.^55^ engineered immunomodulatory microspheres, constituted by biodegradable poly lactic-*co*-glycolic acid polymer, to release IFN-γ within the TME. When delivered via intra-arterial transcatheter injection, these microspheres enabled localized IFN-γ release and significantly enhanced NK cell infiltration in an orthotopic liver cancer model. After adoptive transfer of *ex vivo*-expanded NK cells, mice with *CXCL10*-transfected melanoma showed enhanced NK cell infiltration into tumors, resulting in significantly reduced tumor burden and extended survival compared with those with CXCL10-negative tumors.^56^ In a mesothelioma mouse model, a CXCL11-armored oncolytic vaccinia virus increased intratumoral infiltration of CD8^+^ T cells and, in two of nine mice, NK cell infiltration, resulting in reduced tumor burden and prolonged survival.^57^ We found that expanded NK cells showed significantly enhanced migration toward and infiltration into osteosarcoma engineered to over-secrete CXCL9, -10, and -11 *in vitro* and *in vivo*. In mice with CXCL10-secreting osteosarcoma tumors, infusion of expanded NK cells led to reduced tumor burden compared with those with CXCL10-negative tumors.^53^ These data demonstrated the importance of CXCL9, -10, and -11 in guiding migration and infiltration of CXCR3-positive expanded NK cells into solid tumors. Priming the TME to produce CXCL9, -10, or -11 may be an effective strategy to recruit CXCR3-expressing NK cells and improve the effectiveness of NK cell-based therapies for solid tumors. C-X3-C motif chemokine receptor 1 (CX3CR1), the receptor for C-X3-C motif chemokine ligand 1 (CX3CL1), is also expressed on NK cells. Intratumoral injection of an adenoviral vector expressing CX3CL1 (AdFKN) into colon cancer or melanoma tumors induced secretion of CX3CL1. Administration of AdFKN attracted a substantial number of NK cells to tumor tissues and resulted in striking inhibition of tumor growth.^58^ CCR5, a receptor for CCL3, -4, and -5, is expressed at low levels on expanded NK cells.^59^ Treatment with an irradiated tumor vaccine that secretes CCL3 resulted in a 3-fold increase in the NK cell number within murine colon tumors.^60^ Melanoma cells infected with an arenavirus produced an elevated level of CCL5, which promoted NK cell recruitment and resulted in tumor regression.^61^ Combination strategies, such as transduction of NK cells to overexpress CCR5 alongside delivering a vaccinia virus expressing CCL5 into the TME, also successfully enhanced NK cell infiltration and boosted anti-tumor activity in mice bearing colorectal cancer xenografts.^59^

There is a concern that over-secretion of certain chemokines potentially enhances pro-tumor effects. For instance, CCR5 is also expressed on tumor cells and over-secretion of CCL5 can promote tumor cell survival, proliferation, and metastasis through the CCR5-CCL5 axis.^62^ Silencing the chemokine receptor selectively on tumor cells by an oncolytic virus^63^ or other methods^64^^,^^65^ may be employed to address the potential concern.

### Breaking down the ECM

The dense ECM acts as a physical barrier that limits immune cell penetration into the tumor core. Targeting the tumor stroma directly can help decrease ECM buildup and alleviate solid stress. Collagen and its production can be targeted.^66^^,^^67^ Matrix metalloproteases (MMPs) degrade stromal collagen.^67^ NK cells manufactured to express MMP-8 demonstrated enhanced migration, rejected the tumor, and prolonged survival in NSG mice engrafted with human ovarian cancer.^68^ Suppressing collagen synthesis offers an alternative approach to decrease collagen within tumors. A lysyl oxidase (LOX)-like-2-targeting antibody (simtuzumab) reduces collagen cross-linking.^69^ Losartan, an antihypertensive, inhibits fibroblasts from producing collagen-I.^70^^,^^71^ Enzymes like PEGylated recombinant human hyaluronidase PH20 can degrade hyaluronic acid (HA).^72^ Alternatively, 4-methylumbelliferone can be used to specifically inhibit HA production by fibroblasts.^73^ Heparan sulfate (HS) proteoglycans constitute key structural components of the ECM. Heparanase 1 (HPSE) is the sole enzyme that degrades HS. NK cells genetically modified to express HPSE as an integral membrane protein on their surface exhibited significantly improved infiltration into cancer cell line spheroids and HNSCC xenografts in mice. Consequently, tumor growth was markedly inhibited without inducing any noticeable side effects.^74^

Malignant cells, along with stromal cells in the TME, often secrete proteolytic enzymes such as MMPs and cathepsins to degrade the ECM and pave the way for dissemination.^75^ While ECM degradation may help NK cells infiltrate tumors, it also risks facilitating tumor invasion and metastasis. Therefore, rather than broadly degrading the ECM, more refined strategies are now being developed to manage its structure without promoting malignancy. One such approach selectively targets tumor-specific proteases, like MMP-2 and -9, to reduce ECM degradation associated with metastasis while sparing normal tissue function.^76^ Another strategy focuses on inhibiting enzymes such as LOX, which crosslink and stiffen the ECM, because stiffening is known to support tumor progression.^77^^,^^78^ Inhibiting LOX can, therefore, render the ECM less conducive to invasion. Therapies are also being developed to normalize the ECM rather than dismantle it. For example, enzymatic agents like PEGylated hyaluronidase or TGF-β inhibitors can reduce ECM density and tumor interstitial pressure, enhancing drug or immune cell penetration without compromising residual structural barriers.^79^^,^^80^ Targeting stromal cells, particularly cancer-associated fibroblasts that actively remodel the ECM, also aims to prevent tumors from manipulating their microenvironment to promote spread.^81^ Additionally, physical containment strategies using an artificial ECM are being explored to trap malignant cells and prevent metastasis.^82^ Blocking integrin-mediated interactions between tumor cells and the ECM offers yet another way to hinder invasion without necessitating ECM degradation.^83^ Together, these evolving approaches reflect a strategic shift toward precise modulation of the ECM that supports therapy delivery while minimizing the risk of unintentionally enhancing tumor invasiveness.

Tumors with the minimal ECM reduce the need to break down the ECM, making NK cell-based therapies more feasible and potentially more effective in such tumor types. For instance, medullary carcinomas are characterized by the minimal ECM and prominent lymphoid infiltration.^84^ However, ECM content alone does not fully predict immune accessibility. Some tumors with low ECM content but poor vascularization may still resist immune cell infiltration.^85^ Therefore, both ECM density and tumor vascular features influence the success of NK cell therapies. Overall, tumors with low ECM content and adequate vascularization present the most favorable conditions for NK cell-based treatment strategies.^86^

### Normalizing the tumor vasculature

Solid tumors show dysregulated excessive new blood vessel formation.^87^^,^^88^ Structurally, blood vessels are often dilated and follow a twisted, irregular path, with some regions receiving inadequate perfusion, while others are well-perfused.^89^^,^^90^^,^^91^^,^^92^^,^^93^^,^^94^ Vascular normalization can enhance NK cell entry into the tumor bed by improving blood perfusion throughout the tumor.^95^ Although this increased blood supply might seem to risk fueling tumor growth by providing more oxygen and nutrients, evidence suggests that, when carefully timed and combined with therapies like immunotherapy or chemotherapy, vascular normalization can shift the TME in a way that suppresses tumor progression rather than promoting it.^96^^,^^97^ Vascular endothelial growth factor (VEGF) released by tumor cells binds to VEGF receptor 2 (VEGFR2), thereby promoting vascular growth.^98^ While high-dose VEGF inhibition starves tumors by blocking angiogenesis, lower or optimally dosed anti-VEGF treatment can contribute to vascular normalization.^99^ CAR NK cells that simultaneously targeted programmed death-ligand 1 (PD-L1), EGFR, and VEGFR2 inhibited tumor growth in a lung cancer mouse model.^100^

### Anti-TGF-β therapy

A key challenge in achieving strong NK cell cytotoxicity is their encirclement by immunosuppressive cells and molecules within the TME, creating an immunosuppressive environment that NK cells must overcome. Our genomic analysis in an osteosarcoma xenograft model suggested that upregulation of TGF-β signaling may be a possible mechanism of resistance to treatment with NK cells and an IL-15 agonist NKTR-255.^53^ Acute TGF-β exposure was shown to inhibit IL-15-induced activation of the mechanistic target of rapamycin in NK cells, leading to metabolic inhibition, increased expression of tissue residency-like traits, and loss of anti-tumor effector function.^101^^,^^102^^,^^103^ However, TGF-β resistance, or imprinting, is induced by continuously exposing NK cells to TGF-β and IL-2 for 14 days during their expansion. TGF-β-imprinted NK cells produce higher levels of IFN-γ and tumor necrosis factor-α compared with non-imprinted cells, regardless of the presence or absence of TGF-β. Moreover, TGF-β-imprinted NK cells exhibit greater cytotoxicity than non-imprinted cells and show enhanced resistance to TGF-β-induced reductions in their killing ability.^104^ Additional strategies to improve NK cell-based therapies by reducing circulating TGF-β, blocking ligand-receptor interactions, or inhibiting TGF-β signaling pathways are currently being explored in both preclinical and clinical settings. These include TGF-β neutralizing antibodies, TGF-β receptor (TGF-βR) I kinase inhibitors, SMAD3-silenced NK cells, NK cells engineered with a dominant-negative TGF-βRII, and NK cells modified to express a chimeric receptor combining the extracellular and transmembrane domains of the TGF-βRII with the intracellular domain of the NK cell-activating receptor NKG2D.^105^ DePeaux et al.^106^ developed an oncolytic vaccinia virus engineered to produce a genetically encoded TGF-βRII inhibitor. This inhibitor-expressing vaccinia virus induced tumor regressions in resistant models and demonstrated strong synergy with checkpoint blockade therapy in an extremely aggressive melanoma model. Importantly, this tumor-targeted viral delivery of TGF-β inhibition avoided the autoimmune or other side effects typically linked to systemic TGF-β/TGF-βR blockade.

### Immune checkpoint inhibitors

The TME overexpresses various immune checkpoints including programmed death-1 (PD-1)/PD-L1, T cell immunoreceptor with immunoglobulin and immunoreceptor tyrosine-based inhibitory motif domain (TIGIT) and IL-1 receptor 8 (IL-1R8), which enables the TME to evade immune recognition and elimination.^105^ When used in combination with checkpoint inhibitors, NK cells can be potentiated to improve anti-tumor activity.

Elevated PD-1 expression on NK cells has been observed in gastric cancer, multiple myeloma, Kaposi sarcoma, malignant mesothelioma, and adenocarcinoma^107^ and is also associated with poor survival outcomes in esophageal and liver cancers. PD-1 expression on NK cells results in their functional impairment and promotes tumor immune evasion. *In vitro*, blocking PD-1/PD-L1 signaling significantly increases cytokine production and degranulation, while reducing apoptosis in NK cells isolated from patients with esophageal squamous cell carcinoma (ESCC). Importantly, treatment with a PD-1-blocking antibody significantly suppressed the growth of ESCC xenografts in nude mice, and this tumor suppression was entirely reversed when NK cells were depleted, strongly indicating that PD-1 acts as an inhibitory regulator of NK cells in digestive cancers.^108^ Recently, there has been interest in combining PD-1 or PD-L1 blockade with oncolytic virotherapy,^109^^,^^110^^,^^111^ and these combinations have had preclinical promise.

TIGIT competes with the NK cell-activating receptor DNAX accessory molecule-1 for its shared ligands CD112 (PVRL2) and CD155 (PVR), thereby directly suppressing NK cell cytotoxicity.^112^ *In vitro*, blocking TIGIT enhances the anti-tumor effect of trastuzumab (a recombinant humanized anti-HER2 mAb), which depends on NK cell-mediated antibody-dependent cellular cytotoxicity (ADCC).^113^ Recent studies have demonstrated increased TIGIT expression on tumor-infiltrating NK cells in mouse models of subcutaneously implanted solid tumors, with this elevated TIGIT expression linked to functional exhaustion of NK cells and tumor progression. Blocking TIGIT with mAbs reversed NK cell exhaustion in various tumor models, increased infiltration of activated (CD69^+^) NK cells into tumors, and subsequently improved overall survival.^114^ These findings indicate that the TIGIT signaling pathway in NK cells contributes to tumor immune evasion and that overcoming NK cell exhaustion is essential for the success of anti-tumor immunotherapy.^115^

IL-1R8, also referred to as toll-IL-1R8 or single immunoglobulin IL-1-related receptor, suppresses cell activation mediated by IL receptors and toll-like receptors. It is broadly expressed across various epithelial tissues, particularly in epithelial cells of the kidney, digestive tract, liver, lung, and lymphoid organs, as well as on monocytes, B and T cells, dendritic cells, and NK cells.^116^ Recently, Molgora et al.^117^ identified IL-1R8 as a checkpoint protein in NK cells regulating their anti-tumor activity in solid tumors. Using IL-1R8-deficient (*Il1r8*^−/−^) mice, they observed that NK cells lacking IL-1R8 exhibited significantly higher expression of activating receptors, such as NKG2D, DNAX accessory molecule-1 and Ly49H, and Fas ligand, along with increased production of IFN-γ and granzyme B. Additionally, partial silencing of IL-1R8 in human NK cells using small interfering RNA led to significantly increased IFN-γ production and CD69 expression. In a model of MN/MCA1 sarcoma spontaneous lung metastasis, *Il1r8*^−/−^ mice exhibited a decreased number of hematogenous metastases, and this protective effect was entirely lost when NK cells were depleted in *Il1r8*^−/−^ mice. Furthermore, adoptive transfer of *Il1r8*^−/−^ NK cells significantly decreased both the number and size of lung and liver metastases in mice with MC38 colon cancer, whereas *Il1r8*^+/+^ NK cells showed no effect. These findings indicate that IL-1R8 acts as a negative regulator of NK cells and its inhibition enhances human NK cell effector function.

## Cytokine stimulation

To address the limitations of NK cell therapy including a small number of active NK cells and poor *in vivo* persistence of NK cells, challenges in both hematological malignancies and solid tumors, cytokines have been utilized.

### IL-15

IL-15 promotes NK cell development and survival of mature NK cells.^118^^,^^119^^,^^120^ Culturing peripheral blood mononuclear cells (PBMCs) with K562-based artificial antigen-presenting cells (K562-mbIL15-41BBL), genetically engineered to express the NK-stimulatory molecules membrane-bound IL-15 (mbIL15) and 4-1BB ligand (41BBL), yields a median NK cell expansion of over 1,000-fold after 3 weeks, without promoting T cell growth.^121^ We have demonstrated that *ex vivo*-expanded NK cells with K562-mbIL15-41BBL upregulated activating receptors, including CD69, NKp30, NKp44, and NKG2D.^122^

**Figure 2:**
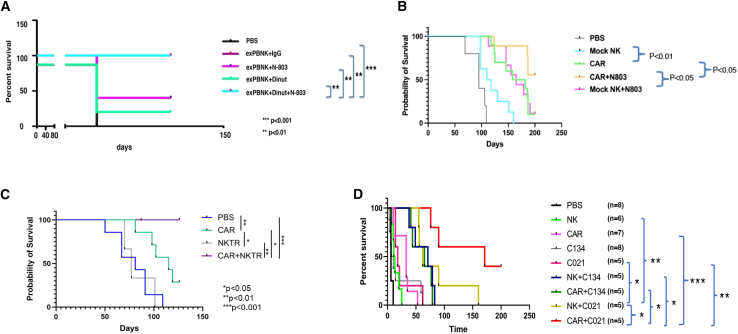
IL-15 or IL-21 stimulation significantly enhanced NK/CAR NK cell anti-tumor activity in xenograft mouse models (A) exPBNK cells combined with Dinut and N-803 significantly extended survival of NSG mice in a U2OS osteosarcoma xenografted model. Adapted from: Chu et al.^127^ Reused with permission from BMJ publishing group. (B) Anti-ROR1 CAR NK cells combined with N-803 significantly extended survival of NSG mice in a CHLA255 neuroblastoma xenografted model. Adapted and reused with permission from: Chu et al.^38^ (C) Anti-MCAM CAR NK cells combined with NKTR-255 significantly extended survival of NSG mice in an SK-N-FI neuroblastoma xenografted model. Adapted and reused with permission from: Luo et al.^39^ (D) Anti-ROR1 CAR NK cells combined with C021 significantly extended survival of NSG mice in a CHLA255 neuroblastoma xenografted model. Adapted and reused with permission from: Chu et al.^143^ Statistical analysis of survival between groups was performed using the log rank test. ∗*p* < 0.05, ∗∗*p* < 0.01, ∗∗∗*p* < 0.001 in (A), (C), and (D). exPBNK, expanded peripheral blood NK; Dinut, dinutuximab; C134, non-cytokine-secreting parental oncolytic herpes simplex virus; C021, C134-based virus modified to overexpress IL-21.

NK cell persistence and function can be enhanced through exogenous IL-15.^123^^,^^124^ Recombinant human IL-15 (rhIL-15)-based immunotherapies have generated significant interest as cancer treatments. However, rapid plasma clearance of rhIL-15 poses a significant limitation in its development as an immuno-oncology therapeutic.^125^ An alternative rhIL-15-based agonist, N-803 was developed to enhance the pharmacokinetic properties of rhIL-15. N-803 is a complex consisting of an amino acid-substituted (N72D) rhIL-15 agonist bound to an IL-15 receptor (IL-15R) sushi domain fused with an IgG1 Fc.^126^ N-803 combined with dinutuximab and *ex vivo*-expanded NK cells markedly enhanced *in vitro* cytotoxicity against GD2-positive pediatric solid tumors and increased survival in NSG mice bearing xenografts (Figure 2A).^127^ Furthermore, N-803 increased both the *in vitro* and *in vivo* anti-tumor activity of anti-ROR1 CAR NK cells targeting neuroblastoma (Figure 2B).^38^ Although N-803 has shown some therapeutic efficacy in clinical trials,^128^^,^^129^ its proliferative effect decreases with repeated dosing, indicating that extended treatment may result in reduced biological responsiveness (tachyphylaxis).^126^ NKTR-255 was designed to enhance the performance of existing rhIL-15-based immunotherapeutics. This innovative polymer-conjugated rhIL-15 was developed to effectively engage the IL-15R complex and sustain prolonged pathway activation. Preclinical studies have demonstrated that NKTR-255 exhibits higher binding affinity to IL-15Rα and reduced *in vivo* clearance compared with rhIL-15 (22 vs. 2.5 h). It has also been shown to trigger STAT5 phosphorylation, reduce tumor burden in a metastatic lung cancer mouse model, and boost NK cell activation and proliferation.^130^ We demonstrated that NKTR-255 significantly enhanced CAR NK cell efficacy against neuroblastoma (Figure 2C)^39^ and Ewing sarcoma^40^ *in vitro* and *in vivo*. Combining NK cells with NKTR-255 further decreased tumor burden and increased survival in mice with CXCL10-positive osteosarcoma tumors.^53^

Another approach to IL-15-based immune modulation of NK cells is armoring NK or CAR NK cells with IL-15. Recent preclinical and clinical studies in hematological malignancies have demonstrated that expression of soluble IL-15 in CAR NK cells targeting CD19 and CD123 led to improved cytotoxicity and persistence *in vivo*.^46^^,^^47^^,^^131^ In solid tumors including colorectal cancer and pancreatic ductal adenocarcinoma, IL-15-secreting anti-CD70 CAR NK cells effectively eliminated both CD70-positive tumor cells and cancer-associated fibroblasts. IL-15 stimulation resulted in upregulation of CAR expression and increased secretion of pro-inflammatory cytokines.^132^

### IL-21

IL-21 also has the ability to enhance the NK cell proliferation and cytotoxic activity.^133^ We have been utilizing membrane-bound IL-21 (mbIL21)-expressing K562 cells (K562-mbIL21-41BBL) for NK expansion.^38^^,^^127^ K562-mbIL21-41BBL expands NK cells out of PBMCs by 30,000–35,000-fold,^134^ circumventing the mechanism of resistance to NK cell therapies secondary to limited numbers of NK cells. These *ex vivo*-expanded NK cells using mbIL21 proved safe and resulted in significantly increased NK cell numbers and functionality, leading to improved survival in patients with myeloid malignancies.^135^^,^^136^

In addition, IL-21 aids in maintaining survival and memory-like responses of NK cells^137^ and plays a crucial role in restoring the function of exhausted NK cells.^138^ In preclinical studies, IL-21 has shown strong anti-tumor activity by activating NK and cytotoxic T cells and stimulating the production of IFN-γ.^139^^,^^140^ Clinical trials in metastatic melanoma^141^^,^^142^ have showcased a favorable safety profile along with significant anti-tumor effects.

Systemic administration of IL-21 may be constrained by its inability to achieve sufficient concentrations throughout the entire tumor. Delivering IL-21 locally via oncolytic viruses provides benefits including minimized systemic toxicity and effective tumor cell lysis. An oncolytic herpes simplex virus was genetically engineered to encode IL-21 (C021) and utilized to infect tumor cells. The virus-infected tumor cells secreted IL-21, which enhanced NK cell activation and proliferation. We demonstrated that combining anti-ROR1 CAR NK cells with C021 markedly decreased tumor burden and prolonged survival in neuroblastoma xenografted NSG mice (Figure 2D). Our finding suggests that C021 modulated the TME and enhanced the therapeutic effectiveness of NK and CAR NK cells.^143^

## NK cell engagers

Bispecific NK cell engagers (BiKEs) and trispecific NK cell engagers (TriKEs) can effectively redirect NK cells toward tumor cells, thereby enhancing their cytotoxic activity. BiKEs simultaneously bind to CD16 on NK cells and a specific epitope on the tumor surface, thereby activating NK cells and leading to tumor cell lysis. A trivalent BiKE directed against HER2 and CD16 exhibited greater potency than an anti-HER2 scFv-Fc fusion protein both *in vitro* and in an ovarian cancer model.^144^ Several additional antibodies that target both HER2 and CD16 have been reported.^145^^,^^146^ Likewise, a BiKE that targets EpCAM and CD16 significantly enhanced ADCC, promoted NK cell degranulation, and increased IFN-γ production against EpCAM-positive cell lines from prostate cancer, breast cancer, colon cancer, and HNSCC.^147^ These BiKEs have been modified to further enhance their effectiveness. A tribody that targets HER2 with two HER2-specific scFvs linked to CD16 [(HER2)2xCD16] demonstrated superior efficacy compared to trastuzumab against HER2-positive breast, pancreatic, ovarian, and esophageal tumor cells, leading to enhanced NK cell degranulation and increased granzyme B release.^148^ TriKEs are designed similarly to BiKEs, but they incorporate a stimulatory cytokine like IL-15 or IL-21. TriKEs improved NK cell survival and proliferation more effectively than BiKEs in NSG mice bearing tumors.^149^ An EpCAM-targeting TriKE containing a modified IL-15 cross-linker improved NK cell proliferation, survival, and ADCC against EpCAM-positive colorectal cancer cells *in vitro*.^150^ Vallera et al.^151^ also designed a TriKE incorporating an IL-15 component and targeting an antigen B7-H3, which is expressed by various solid tumors (cam1615B7H3). cam1615B7H3 improved NK cell activity, proliferation, and targeted killing of various B7-H3-positive human cancer cell lines and led to reduced tumor burden in mice grafted with human ovarian cancer. Similarly, CAM1615HER2 composed of a camelid VHH antibody fragment targeting CD16 and a scFv specific for HER2 linked by IL-15 demonstrated strong *in vitro* cytotoxicity against human ovarian cancer cell lines and induced robust anti-tumor effects in an *in vivo* xenograft model engrafting both human ovarian cancer cells and human NK cells.^152^ The outcomes of preclinical studies are promising, and further clinical development of BiKEs and TriKEs is warranted.

## Other approaches

### Ultrasound-mediated targeted delivery of NK cells

Ultrasound can be used to enhance NK cell infiltration into the tumor site. In a rodent model of human metastatic breast cancer, focused ultrasound has demonstrated potential for targeted delivery of NK cells to the brain. Disrupting the blood-brain barrier with focused ultrasound and microbubbles significantly increased the average NK cell-to-tumor cell ratio.^153^ Alkins et al.^154^ demonstrated that ultrasound-guided targeted delivery of NK cells to the tumor site slowed tumor progression and extended survival in an orthotopic rodent brain tumor model of HER2-amplified human breast cancer.

### Nanoparticle-enabled NK cell immunotherapies

Due to their small size (10–100 nm), nanoparticles are well suited to enhance NK cell-based immunotherapies in ways that traditional antibody delivery methods may not achieve. Nanotechnology could potentially assist NK cells in many ways: boosting NK cell function, improving NK cell homing, delivering RNA interference to enhance NK cell activity, genetically modifying NK cells, and activating the NKG2D receptor.^155^

## Conclusions and future perspectives

**Figure 3:**
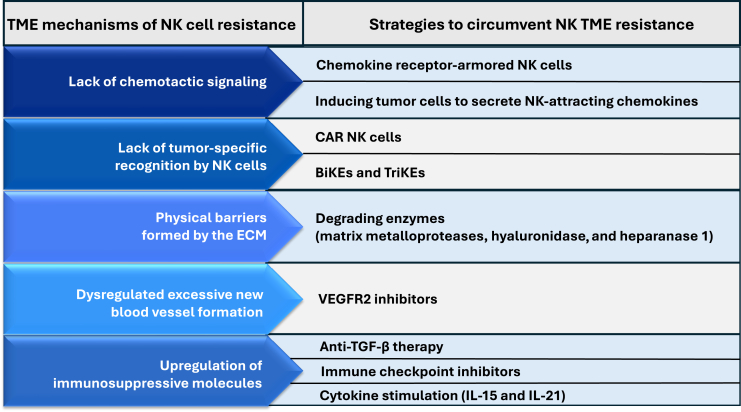
Strategies to circumvent solid tumor microenvironment resistance

Although adoptive NK cell therapies have shown safety and selected efficacy in hematological malignancies, they have not yet produced significant clinical benefits in pediatric or adult solid tumors. This is in part secondary to the lack of chemotactic signaling hindering intratumoral infiltration of NK cells as well as immunosuppressive TME impairing function and persistence of NK cells. Many strategies have been developed to circumvent NK TME resistance (Figure 3). Reversing chemokine/chemokine receptor mismatch can improve NK cell homing and trafficking. Genetic modulation of NK cells to express chemokine receptors has been shown to increase NK homing and infiltration into tumors. Another approach of inducing tumor cells to secrete NK-attracting chemokines initially faced challenges in its clinical application. The use of IFN-γ, which was common in earlier studies, has been limited due to concerns for systemic toxicity. Now, emerging strategies including oncolytic virotherapy and nanoparticles have made modulation of the TME a more attractive approach. Infiltrated NK cells need to persist and maintain their cytotoxic function within the hostile TME in order to exert their maximum efficacy. Inhibition of TGF-β and immune checkpoints and NK stimulation by cytokines have been investigated. As tumors escape immune surveillance through numerous mechanisms, a combination of multiple strategies to enhance chemoattraction while changing the TME from immunosuppressive to immunopermissive may be needed to circumvent obstinate resistance in solid tumors. With recent advances in genetic engineering, an ultimate combination therapy with minimal systemic toxicity can be genetic modification of NK cells to express multiple proteins including chemotaxis receptors, CARs, and ECM-degrading enzymes, while introducing an oncolytic virus to manipulate the TME to secrete NK-attracting chemokines and NK-stimulatory cytokines, inhibit presentation of immune checkpoints, and express TGF-βR inhibitors. In guiding and optimizing such complex therapeutic combinations, artificial intelligence and computational modeling may play a pivotal role by predicting synergistic effects, minimizing toxicity, and personalizing treatment design based on tumor and TME-specific data.

## Acknowledgments

This work was primarily supported by a grant from the 10.13039/100000054National Cancer Institute 10.13039/100015338Cancer Moonshot U54 CA232561 (M.S.C.). Additional support was received from the 10.13039/100006058St. Baldrick's Foundation (M.S.C.), 10.13039/100000902Pediatric Cancer Research Foundation (M.S.C.), and 10.13039/100029695Children's Cancer Foundation (M.S.C.). We would like to acknowledge Virginia Davenport, RN, and Erin A. Morris, RN, BSN, at the Department of Pediatrics, New York Medical College, in preparation and submission of this manuscript. The graphical abstract and Figure 1 were created in BioRender.

## Author contributions

The first draft of the manuscript was written by S.E., W.L., and M.S.C. H.Z., J.M.R., and A.R.C. contributed intellectually. All authors reviewed and approved the final manuscript.

## Declaration of interests

M.S.C. has served as a consultant for Jazz Pharmaceuticals, Omeros Pharmaceuticals, and AbbVie; in the Speakers Bureau for Jazz Pharmaceuticals and Amgen; and received research funding from Merck, Miltenyi Biotec, Servier, Omeros, Jazz, and Janssen. The other co-authors have no relevant financial or non-financial interests to disclose.
